# Circulating Type I Interferon Levels and COVID-19 Severity: A Systematic Review and Meta-Analysis

**DOI:** 10.3389/fimmu.2021.657363

**Published:** 2021-05-12

**Authors:** Rafaela Pires da Silva, João Ismael Budelon Gonçalves, Rafael Fernandes Zanin, Felipe Barreto Schuch, Ana Paula Duarte de Souza

**Affiliations:** ^1^ Biomedical Graduate Course, School of Health and Life Science, Pontifical Catholic University of Rio Grande do Sul, Porto Alegre, Brazil; ^2^ Laboratory of Clinical and Experimental Immunology, School of Health and Life Science, Pontifical Catholic University of Rio Grande do Sul, Porto Alegre, Brazil; ^3^ Department of Health and Human Development, La Salle University, Canoas, Brazil; ^4^ Department of Sports Methods and Techniques, Federal University of Santa Maria, Santa Maria, Brazil

**Keywords:** COVID-19, severity, biomarker, type I interferon, IFN-α

## Abstract

**Introduction:**

Coronavirus disease 2019 (COVID-19) is caused by severe acute respiratory syndrome coronavirus 2 (SARS-CoV-2) infections, resulting in a range of clinical manifestations and outcomes. Laboratory and immunological alterations have been considered as potential markers of disease severity and clinical evolution. Type I interferons (IFN-I), mainly represented by IFN-α and β, are a group of cytokines with an important function in antiviral responses and have played a complex role in COVID-19. Some studies have demonstrated that IFN-I levels and interferon response is elevated in mild cases, while other studies have noted this in severe cases. The involvement of IFN-I on the pathogenesis and outcomes of SARS-CoV-2 infection remains unclear. In this study, we summarize the available evidence of the association of plasma protein levels of type I IFN with the severity of COVID-19.

**Methods:**

The PRISMA checklist guided the reporting of the data. A systematic search of the MEDLINE (PubMed), EMBASE, and Web of Science databases was performed up to March of 2021, looking for articles that evaluated plasma protein levels of IFN-I in mild, severe, or critical COVID-19 patients. Comparative meta-analyses with random effects were performed to compare the standardized mean differences in plasma protein levels of IFN-I of mild versus severe and mild versus critical patients. Meta-regressions were performed to test the moderating role of age, sex, time that the IFN-I was measured, and limit of detection of the assay used in the difference between the means.

**Results:**

There was no significant difference in plasma levels of IFN-α when comparing between mild and severe patients (SMD = -0.236, 95% CI -0.645 to 0.173, p = 0.258, I2 = 82.11), nor when comparing between patients mild and critical (SMD = 0.203, 95% CI -0.363 to 0.770, p = 0.481, I2 = 64.06). However, there was a significant difference between healthy individuals and patients with mild disease (SMD = 0.447, 95% CI 0.085 to 0.810, p = 0.016, I2 = 62.89).

**Conclusions:**

Peripheral IFN-α cannot be used as a severity marker as it does not determine the clinical status presented by COVID-19 patients.

## Introduction

Coronavirus disease 2019 (COVID-19) is caused by severe acute respiratory syndrome coronavirus 2 (SARS-CoV-2) infections, which has already affected more than 134,525,543 people in 217 countries and territories (1). COVID-19 has been considered a pandemic since March 11, 2020, causing more than 2 million deaths globally. SARS-CoV-2 can be transmitted from person to person through droplets from breathing, coughing, sneezing, talking, or through direct contact with contaminated individuals or objects and surfaces (2). The average incubation period is 5 days (3), and the most prevalent signs and symptoms are fever, cough, headache, myalgia, fatigue, gastrointestinal symptoms, and dyspnea (4, 5). COVID-19 causes distinct clinical manifestations among affected individuals, ranging from asymptomatic cases to cases with mild, moderate, severe, or critical symptoms, where they might progress to an acute respiratory distress syndrome (SARS) or even to dysfunction of multiple organs, complications that lead to death (6, 7). The risk of developing a more aggressive disease condition is influenced by age and the presence of comorbidities, such as diabetes, hypertension, obesity, and cardiovascular diseases (2, 8). Markers, such as the increase in C-reactive protein (CRP), D-dimer, and prothrombin time, are important to predict the outcome of the disease (9, 10). Also, immunological disorders were observed, such as leukopenia (11, 12), as well as a significant increase in serum cytokines levels, such as IL-6, TNF-α, and IL-8 (12, 13). Among the altered cytokines, interferons present conflicting results (14).

Interferons are divided into type I (IFN-α and IFN-β), type II (IFN-γ), and type III (IFN-λ) and have a fundamental role in the innate immune system, being part of the first line of defense against viral infections (15). In humans, the type I IFN (IFN-I) family consists of multi-genes that encodes 13 partially homologous IFN-α subtypes (IFN-α1, α2, α4, α5, α6, α7, α8, α10, α13, α14, α16, α17, and α21) and genes that encodes IFN-β, IFN-ε, IFN-κ, and IFN-ω (16, 17). Among the IFN-α2 present subvariants, the best described are α2a and α2b (18). Type I interferons (IFN-I) act as inhibitors of viral replication in infected cells and have a defensive action in uninfected cells. IFN-I can affect dendritic cells, B, T, and NK cells function, enhancing immune response. Most infected cell types can produce IFN-β, but IFN-α is mainly produced by hematopoietic cells (19, 20). Plasmacytoid dendritic cells (pDCs) are considered as a major producer of IFN-α (21). These cells detect viruses through TLR7 and TLR9, inducing secretion of IFN-I through the MyD88-IRF7 signaling pathway (22, 23).

IFN-I is produced rapidly after virus infection and exhibits antiviral activity by binding to its receptor IFNAR and inducing the expression of the interferon-stimulated genes (ISGs) (24). ISGs play an important role in viral infection, presenting an inhibitory effect at different stages of viral replication, such as viral membrane fusion and viral gene expression. They are responsible for the direct antiviral effects or molecules that can regulate IFN-I induction and signal (24–27). Some ISGs can control cell death and metabolism, RNA degradation, and protein synthesis. The IFN-I response can recruit several immune cells and lead to inflammatory disease, and this can develop beneficial or detrimental outcomes for the host (17). Despite its great importance in the antiviral response, the role of IFN-I in COVID-19 is complex (28–30); some studies have suggested a protective role for IFN-I during infection (31, 32), while other studies suggested a deleterious role for IFN-I in COVID-19 (33, 34).

Aging impairs the IFN-I production in pDCs (35, 36). Monocytes from the elderly exhibited impaired RIG-I signaling due to the absence of TRAF3, reducing induction of IFN-I production (37). Also, pDC from healthy males produces less IFN-I after stimulation comparing to females (38). These findings might explain why the elderly are more susceptible to complications from the SARS-CoV-2 infection (39, 40) and the higher prevalence of COVID-19 in men (41). In contrast, smoking and body mass index (BMI) also affect the expression of IFN-I but increasing it, and these two factors tend to worsen the clinical course of the SARS-CoV-2 infection (42). Also, the response to IFN-I was initially considered an inducer of the virus receptor, the angiotensin-converting enzyme 2 (ACE2) (43), which could worsen the patient’s condition. However, it has recently been shown that interferon induces an ACE2 isoform, which does not function as a virus receptor (44).

IFN-I is used to treat various diseases, including hepatitis C (45, 46) and B (47), multiple myeloma (48), polycythemia Vera (49), and are also tested for the treatment of COVID-19. Studies carried out with inhaled or intramuscularly administered IFN-α2b have demonstrated that treatment can accelerate viral clearance and reduce circulating IL-6 and CRP levels (50), in addition to preventing disease progression (51). IFN-β-1b used subcutaneously effectively decreased the time to clinical improvement in severe COVID-19 patients (52). However, IFN-β used subcutaneously did not reduce mortality in patients with COVID-19 in a large clinical study conducted by WHO in 30 countries (53). Conversely, inhaled IFN-β1a improved the recovery time for patients infected with SARS-CoV-2 (54). In a descriptive review of literature about the role of IFN-I in treating COVID-19, it is highlighted that IFN-α use at the early phase of COVID-19 presents a positive outcome, but IFN-β-1a and -1b are more effective to hinder COVID-19 (55). A systematic review and meta-analysis have been previously performed to evaluate the effects of IFN-I and Janus Kinase-inhibitors as treatments for COVID-19 patients and their effectiveness to produce positive outcomes (56). They found that IFN-I treatment is associated with positive clinical outcomes regarding mortality and discharge (56). The time to start IFN-I treatment might be crucial (57).

Disagreements related to the role of IFN-I in patients with COVID-19 demonstrate the importance of further studying these cytokines in the disease. The importance of IFN-I in the pathogenesis of the COVID-19 had been previously reviewed (30, 57–61). Nonetheless, the available evidence regarding the circulating levels of IFN-I and its response and the association with the severity of the disease has not been meta-analyzed. This review aims to integrate the different studies on this subject to verify whether the increase or decrease levels of IFN-I in serum/plasma from patients with COVID-19 is linked to the disease severity.

## Methodology

### Search Strategy

The articles search was carried out by two reviewers (RPS and JIBG), from August 25 to March 21, 2021, in three different bibliographic databases - MEDLINE (PubMed), EMBASE, and Web of Science. The terms used were (“COVID-19” OR “SARS-COV-2” OR “Coronavirus Infections”) AND (“IFN” OR “Interferon type I” OR “Interferon-alpha” OR “Interferon-beta”). Additional research was also carried out on the references of the articles that had the full reading in the second screening phase, and searching in the Research Gate and medRxiv, using the same terms listed above, looking for studies not yet peer-reviewed.

### Inclusion and Exclusion Criteria

All articles that performed comparisons between plasma protein levels of IFN-I (β, α, and subtypes) in patients with different degrees of COVID-19 severity (mild, severe, and critical) were included. Studies that did not present the mean and standard deviation values or errors in the text or figures were excluded. Review articles and meta-analyses were excluded.

### Data Extraction

Authors, publication date, study design, number of participants included (healthy, mild, severe, and critical), mean age, gender, symptoms related to COVID-19, criteria used for classification severity of the disease, comorbidities, IFN-I levels in plasma/serum, methodology for IFN-I analysis, and days after infection that IFN-I was measured were extracted from each of the selected articles. The data of IFN- I levels presented in the format of a graph were extracted using the Graph Grabber software (62).

### Quality Assessment

The methodological quality of the studies was evaluated with the Newcastle-Ottawa scale (63). The quality score of the case-control studies was calculated according to three aspects: the selection of cases and controls (0–4 stars); the comparability of cases and controls (0–2 stars); and the outcomes evaluation (0–3 stars). The maximum score is nine stars, representing high methodological quality.

### Statistical Analysis

Comparative meta-analyses of plasma protein levels of IFN-α were performed between patients with mild versus severe and mild versus critical COVID-19 and between healthy individuals versus patients with mild COVID-19 and healthy individuals versus patients with severe COVID-19. Due to clinical heterogeneity, the use of meta-analyses of random effects was established a priori. The effects of individual studies were calculated using the standardized mean difference (SMD) and 95% confidence interval (95% CI). Effect sizes are considered weak if > 0.2, moderate if > 0.5, or strong if > 0.8 (64). The heterogeneity of effects between studies was calculated using the I² test (65). Meta-regressions were performed to explore whether age, sex, time that the IFN-I was measured, and limit of detection of the assay used are moderators of the difference between different groups. Subgroup analyzes were performed to verify how much the different measuring techniques used influence heterogeneity. Finally, the Egger (66) and Begg-Mazumbar (67) regression tests were performed, together with the visual analysis of the Funnel plot, to assess the presence of publication bias.

## Results

### Search Results

**Figure 1:**
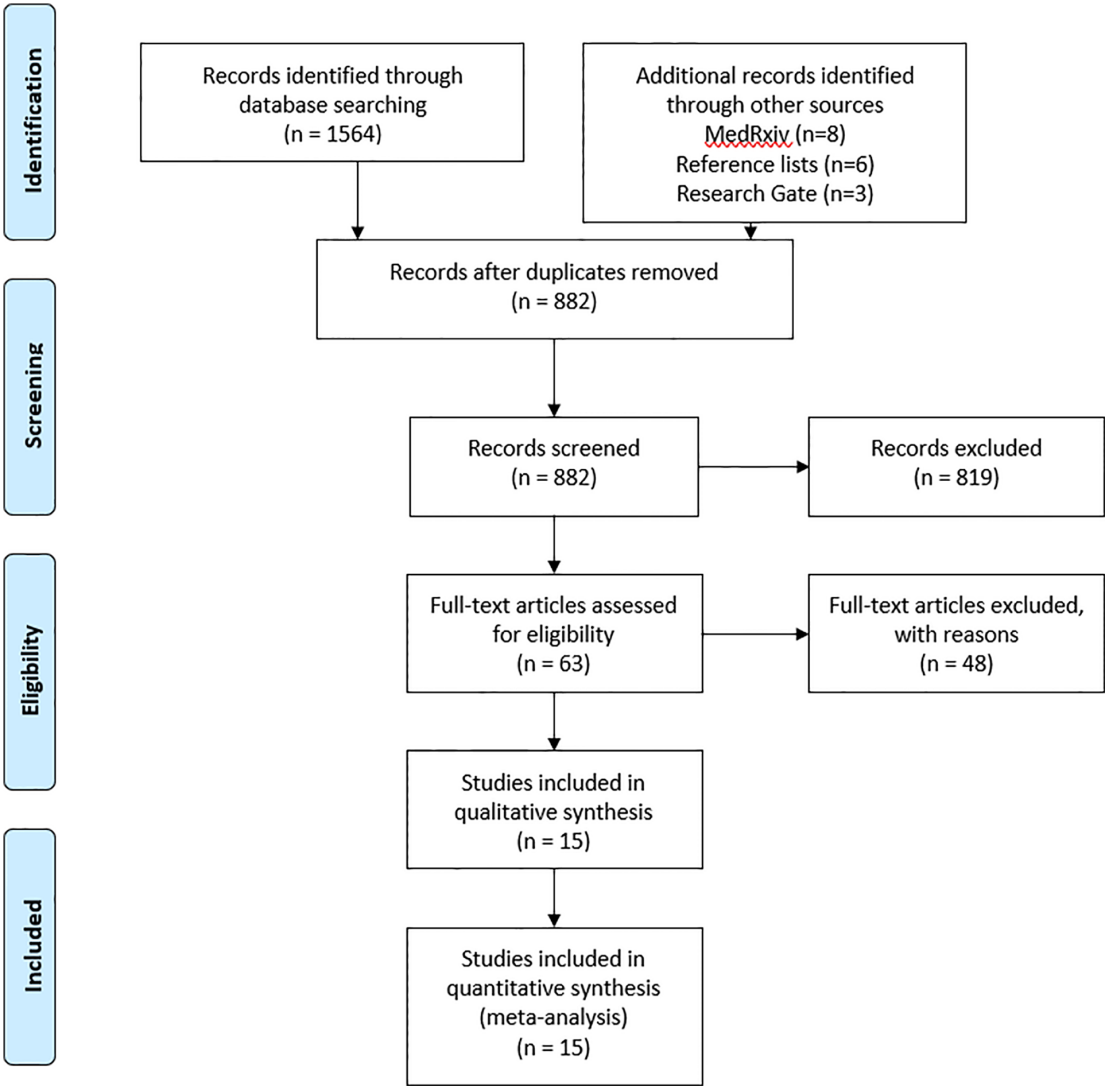
Flowchart of article selection.

The searches of the databases resulted in 1564 articles. Another 17 articles were added manually through searches on Research Gate and medRxiv as they have potentially relevant titles and abstracts. After removing 699 duplicates, 819 articles were excluded for their titles and abstracts. Afterward, 63 studies were read entirely. Of these, 15 studies were included in the meta-analysis (68–81). **Figure 1** represents the flowchart of the meta-analysis containing the details of the articles excluded in each of the stages, including the reason for their exclusions. A complete table listing those articles excluded after an evaluation is given **Supplementary Material 1**.

### Description of Studies and Participants

The measurement of IFN-α was presented in all studies, while of IFN-β in only two of them. The methods used for measuring IFN-α were ELISA, single molecular array (Simoa), Luminex magnetic bead, electrochemiluminescent, bead-based immunoassay for flow cytometry, and microfluid immunoassay fluorescence detection. The average age of participants was 43 to 63 years, and the percentage of male participants ranged from 42 to 83%. The comparison between mild and critical was performed in four of the studies, between mild and severe in 15 studies and between healthy and mild and healthy and severe in nine studies. The cases were considered mild to moderate when presenting fever, myalgia, fatigue, diarrhea, signs of pneumonia on chest CT scan, and requirement of up to 3 L/min of supplemental oxygen to maintain more than 92% SpO2 (68–76). Severe cases were characterized by the need for admission to the intensive care unit (ICU) due to breathing difficulties, needing more than 3 L/min of oxygen, and partial pressure of arterial oxygen (PaO2)/fraction of inspired oxygen (FiO2) ≤ 300 mmHg (69–74, 76, 77). Critically ill patients had respiratory failure, septic shock, and/or multiple organ dysfunction or failure (70–75, 77). **Table 1** contains the main characteristics of the selected studies.

**Table 1:** Main characteristics of the selected articles.

Authors	N (% men)	Mean Age	Kit Source	Methodology	IFN-I Subtype	Time (days)	Limit of detection (pg/ml)	Number of participants that IFN was measured (detected)
Arunachalam et al.	76 (58%)	55	Simoa Technology	Single molecular array	IFN-α	0-40	0.003	58 (57)
Chi et al.	70 (56%)	43.4	Bio-Plex Pro Human Cytokine Screening 48-plex panel (BioRad)	Luminex magnetic bead	IFN-α2	11	0.49	34 (34)
Galani et al.	32 (69%)	63	High sensitivity ELISA (Abcam)	ELISA	IFN-α	01-03	1.0	26 (26)
Hadjadj et al.	50 (58%)	55	Simoa Technology (Quanterix)	Single molecular array	IFN-α2	10	0.003	50 (50)
Henry et al.	52 (57.7%)	51	U-Plex assay (MSD)	Electro chemiluminescence	IFN-α2a	7	4.0	52 (52)
Kwon et al.	31 (42%)	50	Cytometric bead array (BD Bioscience)	Bead-based immunoassay for Flow cytometry	IFN-α	05-10	1.5	31 (8)
Liu et al.	12 (66.7%)	62.5	BioPlex Pro Human Cytokine Screening Panel (BioRad)	Luminex magnetic bead	IFN-α2	07-10	0.49	20 (20)
Lucas et al.	113 (46%)	62.9	Human Cytokine Array/ Chemokine Array 71-403 Plex Panel (HD71)	Luminex magnetic bead	IFN-α2	10	6.56	221 (u)
Ruetsch et al.	151 (66%)	51	Ella custom-design cartrigge (Protein Simple)	Microfluid immunoassay fluorescence detection	IFN-α	–	0.39	110 (0)
Sánchez-Cerrillo et al.	64 (57.8%)	61	Human IFN-α all subtypes Serum ELISA kit (pbl assay science)	ELISA	IFN-α	8	1.95	64 (10)
Silvin et al.	158 (44%)	53	Ultra-sensitive assay S-plex Human IFN-α2a kit (MSD)	Electro chemiluminescence	IFN-α2a	–	0.0049	83 (83)
Tincati et al.	40 (83%)	61	Cytometric bead array (BD Bioscience)	Bead-based immunoassay for Flow cytometry	IFN-α	7	1.5	40 (u)
Turnbull et al.	63 (49.3%)	55.6	Cytokine Human Magnetic 35 Plex Panel, (ThermoFisherScientific)	Luminex magnetic bead	IFN-α	9	30	54 (u)
Thwaites et al.	471 (64.9%)	58.4	Simoa Technology (Quanterix)	Single molecular array	IFN-α	9	0.003	52 (52)
Yang et al.	93 (60%)	46.4	Human Th1/2 cytokine kit II (ACEA NovoCyte, Guangzhou, China)	Bead-based immunoassay for Flow cytometry	IFN-α	–	–	93 (93)

u, unavailable.

### Quality Assessment

The general methodological quality of the studies included in this review was high, with all the studies presenting scores on the Newcastle-Ottawa scale ranging from 7 to 8, demonstrating good methodological quality (**Table 2**).

**Table 2:** Quality assessment of studies using the Newcastle-Ottawa scale.

Studies	Selection	Comparability	Exposure	Total
Arunachalan et al.	3	2	2	7
Chi et al.	4	2	2	8
Galani et al.	4	2	2	8
Hadjadj et al.	4	2	2	8
Henry et al.	4	2	1	7
Kwon et al.	4	2	2	8
Liu et al.	4	2	2	8
Lucas et al.	4	2	2	8
Ruetsch et al.	3	2	2	7
Sánchez-Cerrillo et al.	4	2	2	8
Silvin et al.	4	2	2	8
Tincati et al.	4	2	2	8
Turnbull et al.	4	2	2	8
Thwaites et al.	3	2	2	7
Yang et al.	4	2	1	7

### Meta-Analysis

**Figure 2:**
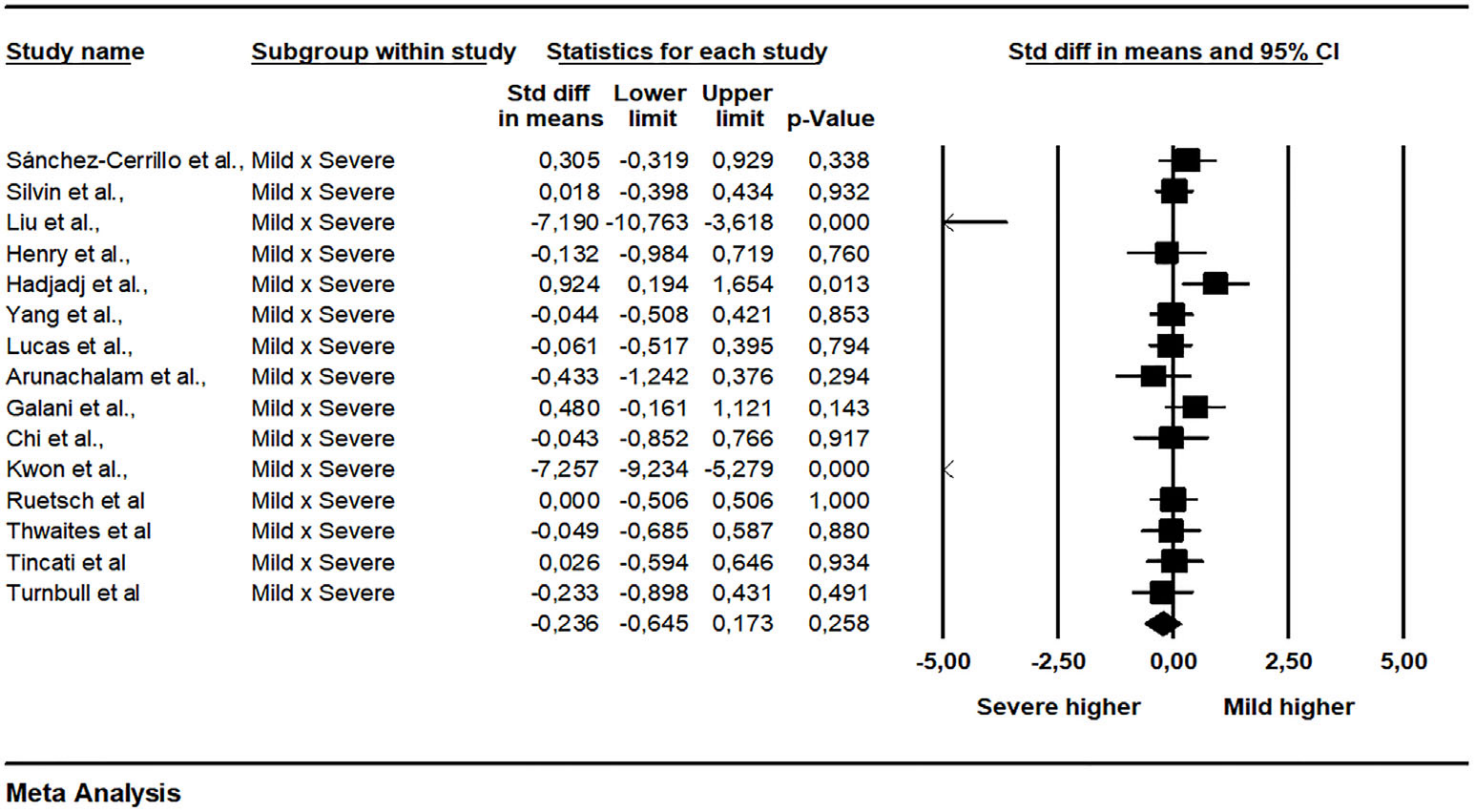
Comparison of plasma IFN-α concentration between mild and severe patients.

**Figure 3:**
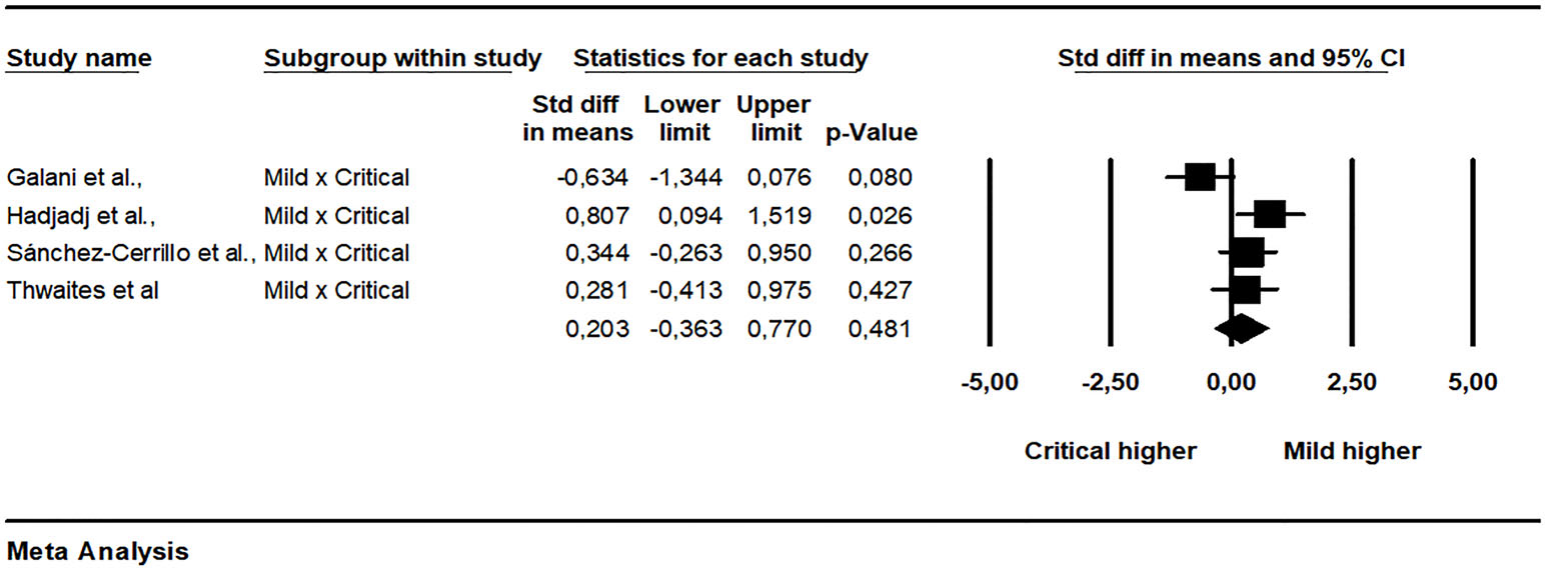
Comparison of plasma IFN-α concentration between mild and critical patients.

**Figure 4:**
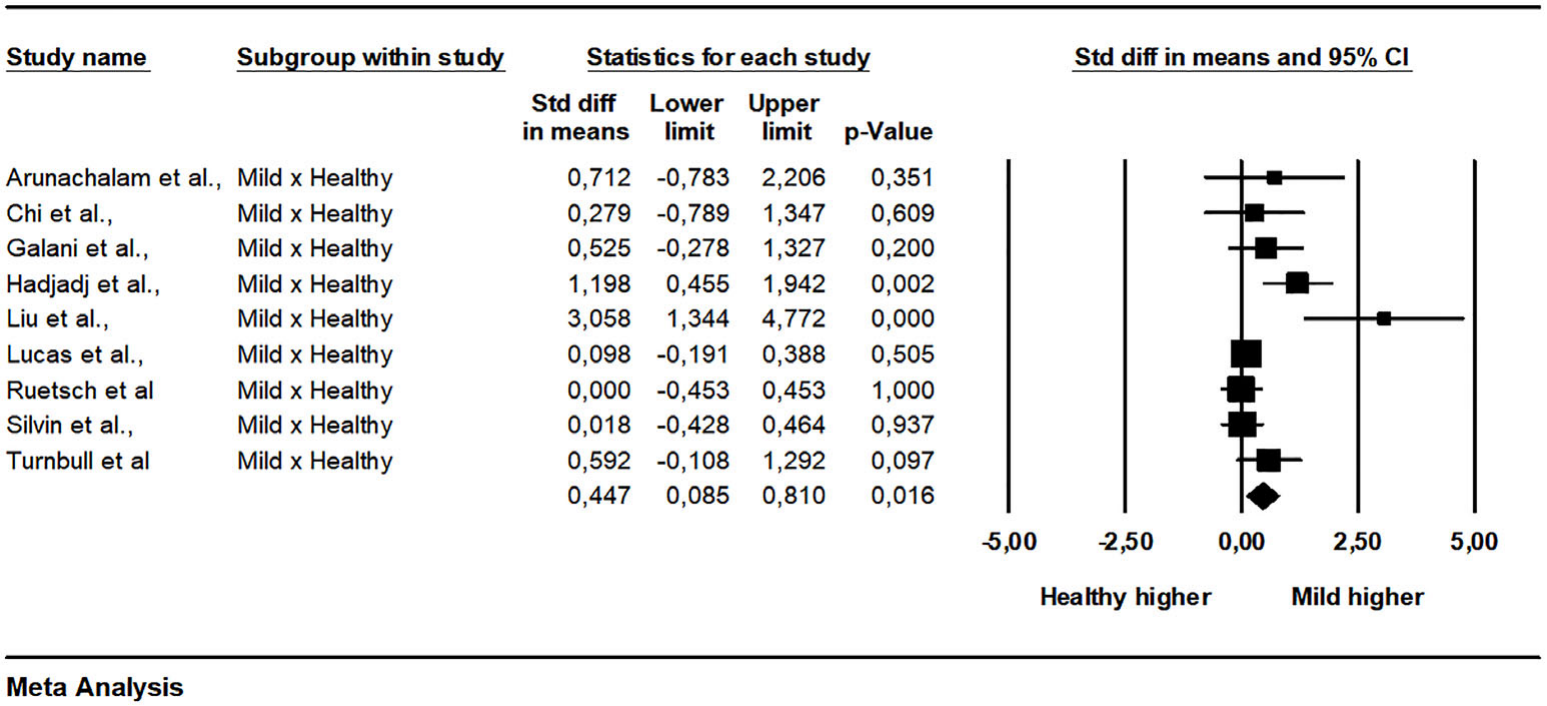
Comparison of plasma IFN-α concentration between mild patients and healthy individuals.

**Figure 5:**
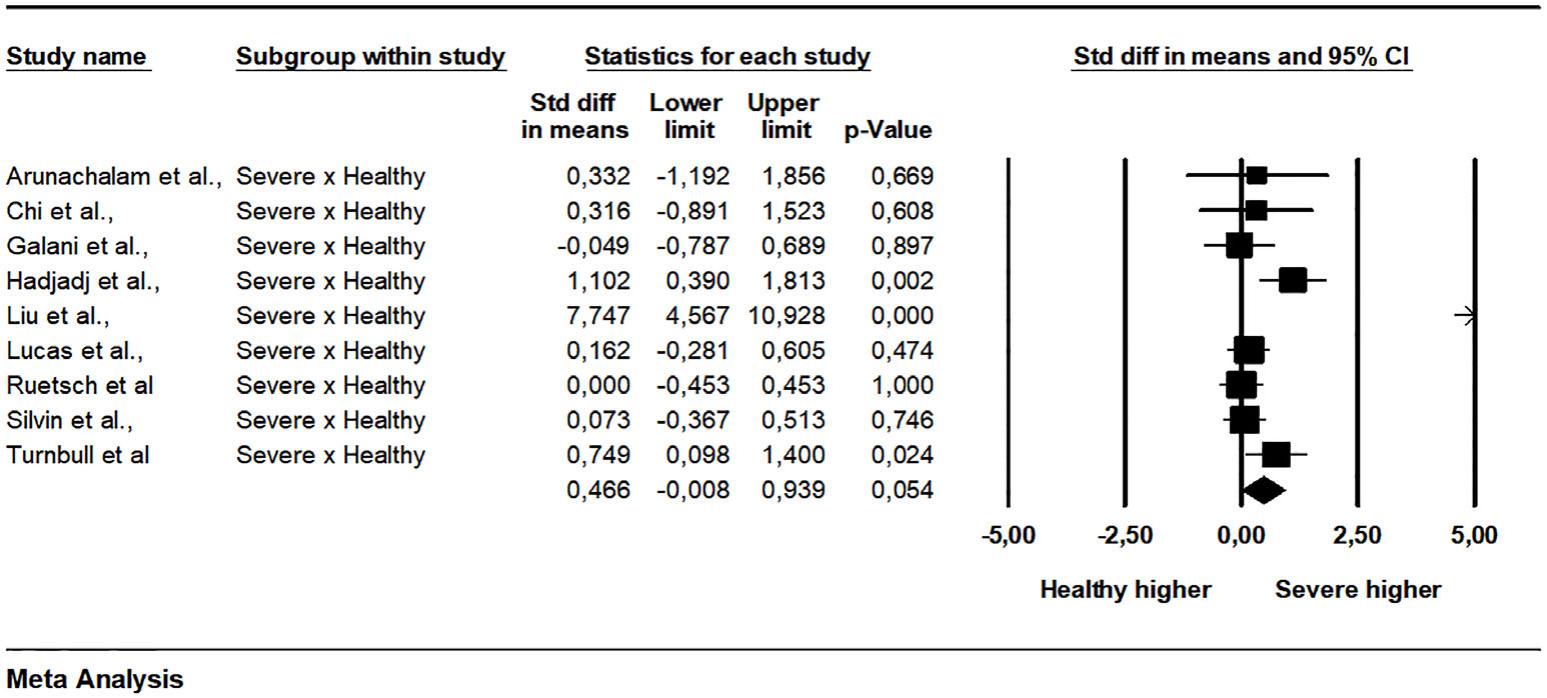
Comparison of plasma IFN-α concentration between severe patients and healthy individuals and.

The pooled data from 15 studies showed a non-significant difference between patients with mild and severe disease (SMD = -0.236, 95% CI -0.645 to 0.173, p = 0.258, I2 = 82.11) (**Figure 2**). A subgroup analysis was made exploring how much the analysis technique explains heterogeneity. When the single molecular array (Simoa) was used there was a significant difference in the analysis (p = 0.001). The Begg-Mazumdar (Tau = -0.28, p = 0.137) and Egger tests did not indicate publication bias (interception = -4.35, p = 0.006). The meta-regression analyzes did not indicate the existence of moderation of the effects by sex (coefficient = 0.0170, 95% CI -0.0080 to 0.0421, p = 0.1818, R² = 0.00), age (coefficient = 0.0058, 95% CI -0.0184 to 0.0300, p = 0.6395, R² = 0.00), limit of detection of the assay used (coefficient = 0.0020, 95% CI -0.0575 to 0.0615, p = 0.9476, R² = 0.00), and time after infection that IFN-I was measured (coefficient = 0.0400, 95% CI -0.1057 to 0.1857, p = 0.5906, R² = 0.00). Between mild and critical, with data grouped from four studies, the difference was also not significant (SMD = 0.203, 95% CI -0.363 to 0.770, p = 0.481, I2 = 64.06) (**Figure 3**). The pooled data from nine studies showed a significant difference between healthy individuals and patients with mild disease (SMD = 0.447, 95% CI 0.085 to 0.810, p = 0.016, I2 = 62.89) (**Figure 4**). Between healthy individuals and patients with severe disease the difference was not significant (SMD = -0.466, 95% CI -0.939 to 0.008, p = 0.054, I2 = 74.73) (**Figure 5**).

## Discussion

Our results indicate that the circulating levels of IFN-α alone are not determinant in the clinical status of patients affected by COVID-19. We found a significant difference in the plasma protein levels of IFN-α between healthy individuals and mild to moderate cases of COVID-19, which is expected since IFN-α increases during viral infection. Most of the studies included in our meta-analysis, which analyzed healthy controls and COVID-19 patients, found differences in the IFN-α levels. However, we compared the data from severe patients, and no significant differences were found between the groups. To our knowledge, this is the first systematic review and meta-analysis associating the circulating levels of IFN-I with the COVID-19 severity.

Regarding the susceptibility to severe COVID-19, Beck et al. (61), in a qualitative review of the literature, provide the perspective that impaired type I interferon response might be a hallmark of severe COVID-19. Still, our findings provide insight that this is not the case, at least for IFN-α circulating levels. However, they also described studies that did not fit our inclusion criteria, such as the study of Zhang et al. (82), that reported in a large genetic sequencing effort to define risk factors to SARS-CoV-2 infection an association with defects in genes of TLR3 and IRF7 – dependent induction and amplification of IFN-I. Also, these authors highlighted the study of Bastard et al. (31), who found the presence of autoantibodies against interferons, including IFN-α, in at least 10% of patients with life-threatening COVID-19. These autoantibodies lead to a plasma reduction of IFN-I, facilitating the viral cycle since the host’s response against it is weakened and increasing the chances of the patient manifesting the severe or critical form of the disease (31). In line with this hypothesis, another article that could not be included in our meta-analysis due to its study design, carried out by Trouillet-Assant et al. (32), assumed that patients who have a decreased plasma concentration of this cytokine have a poor prognosis, whereas patients with the lowest IFN-I concentration have longer stay in the ICU, strengthening the crucial role of IFN-I in antiviral responses.

Another point is that we did not meta-analyze studies measuring IFN-β levels because only a few studies performed these analyses. IFN-I concentrations are tightly regulated and normally difficult to be measured. Hadjadj et al. demonstrated that patients affected by COVID-19 present undetectable IFN-β gene expression and circulating protein in plasma (70). Additionally, an impaired IFN-I response can occur due to the evasion mechanisms of SARS-CoV-2, which allows the virus module to host the immune response. Among these mechanisms is the inhibition of IFN-I production through non-structural protein, such as nsp6, the inhibition of IFN-I signaling through nsp1 (83), and the possible antagonism by nsp13 and the accessory protein ORF6 (84), and more recently, the role of M protein in inhibiting RIG/MDA signaling (85).

Further studies addressed the role of IFN-I during SARS-CoV-2 infection, analyzing IFN-I and ISGs gene expression using molecular approaches, particularly single-cell RNA sequencing analysis, which was also not included in our meta-analysis (86–88). Studies carried out with single-cell RNA sequencing determined a hyperactivation of the IFN-I signaling pathways in critically ill patients, contributing to immune dysfunction, leading to exaggerated reactions that can damage tissues and impair the patient’s evolution (88, 89) mainly at the pulmonary level. This was observed in post-mortem analyzes performed on patients who die due to COVID-19, as they had a high concentration of IFN-I in lung tissues (90). Also, Menezes et al. investigated the interferon, I, II, and III gene expression at the nasal mucosa of COVID-infected patients. These authors found that higher levels of the IFNB1 transcript predicted poor outcomes (91). However, children with COVID-19 present higher levels of IFN-α in nasal fluid than adults and present less severe symptoms, indicating a good outcome (92).

The immunopathogenic role of IFN-I also occurs when an infection has a prolonged duration (93). IFN-I’s role in exacerbating pulmonary inflammation is due to the recruitment of NK cells, T cells, dendritic cells, monocytes, and macrophages through the release of chemokines, such as CCL2 and CXCL10 (60). IFN-I also potentiates the inflammatory response induced by TNF-α and IL-1β in severe disease progression (90), and IFN-α may be the main cytokine mediating these responses (94). A late release of IFN-I in response to SARS-CoV-2, in addition to a superabundant response of the innate immune system, capable of a pro-inflammatory reaction with immunopathogenic potential, might be the reason for a poor outcome (73, 95). The IFN-α role in the COVID-19 might depend on the instant when the initial response was launched, the possible interferences in that response, such as the mechanisms of viral evasion and genetic host mutations, and the duration of the infection.

### Limitations

The present study has some limitations. First, only a small number of articles are included since the subject is recent and does not have many studies exploring the theme in depth. Second, high heterogeneity was found among the studies analyzed and due to the small number of studies. Another limitation is the low sensitivity of the assays used in some studies to measure IFN-α, which might be why IFN-α has not been detected in many cases. An ultrasensitive method is recommended to measure the circulating levels of IFN-I. Although the progression of the infection might affect the levels of IFN-α, in our study, this parameter did not have a significant influence, as demonstrated by the meta-regression analysis.

## Conclusion

With the current systematic review and meta-analysis, it is possible to conclude that the plasma protein levels of type I IFN, based on peripheral measurement of IFN-α, do not demonstrate significant differences between mild, severe, or critical patients. Therefore, IFN-α cannot be used alone as a severity marker for COVID-19.

## Data Availability Statement

The raw data supporting the conclusions of this article will be made available by the authors, without undue reservation.

## Author Contributions

RS performed the database search, extracted the data and wrote the manuscript. JB performed the database search as a second reviewer. FS carried out the meta-analysis and revised the manuscript. AS idealized the hypothesis, revised the database search and the manuscript. RZ performed the final review of the manuscript. All authors contributed to the article and approved the submitted version.

## Funding

FAPERGS – Edital emergencial COVID-19 processo 20/2551-0000258-6. Capes – Financial Code 1 and CNPq.

## Conflict of Interest

The authors declare that the research was conducted in the absence of any commercial or financial relationships that could be construed as a potential conflict of interest.
