# Supplementary material for: Circulating Type I Interferon Levels and COVID-19 Severity: A Systematic Review and Meta-Analysis
Source: Front Immunol. 2021 May 12;12:657363. doi: 10.3389/fimmu.2021.657363 (PMC8149905; doi:10.3389/fimmu.2021.657363)
Supplement: Supplementary file 1 [file DataSheet_1.docx]

Supplementary Material

# Supplementary Data

## Reasons for article exclusion.

### Measuring IFN-Y

Kroemer, M., Spehner, L., Vettoretti, L. et al. COVID-19 patients display distinct SARS-CoV-2 specific T-cell responses according to disease severity. *J Infect*. 2020;4816. doi:10.1016/j.jinf.2020.08.036

Burke, H., Freeman, A., Cellura, D.C. *et al.* Inflammatory phenotyping predicts clinical outcome in COVID-19. *Respir Res* **21,**245 (2020). <https://doi.org/10.1186/s12931-020-01511-z>

Wang F, Hou H, Luo Y, et al. The laboratory tests and host immunity of COVID-19 patients with different severity of illness. *JCI Insight*. 2020;5(10):e137799. doi:10.1172/jci.insight.137799

Liu, J., Li, S., Liu, J., et al. Longitudinal characteristics of lymphocyte responses and cytokine profiles in the peripheral blood of SARS-CoV-2 infected patients. *EBioMedicine*. 2020;55:102763. doi:10.1016/j.ebiom.2020.102763

Zhao, Y., Qin, L., Zhang, P. et al. Longitudinal COVID-19 profiling associates IL-1RA and IL-10 with disease severity and RANTES with mild disease. *JCI Insight*. 2020;5(13):e139834. doi:10.1172/jci.insight.139834

Han, H., Ma, Q., Li, C. et al. Profiling serum cytokines in COVID-19 patients reveals IL-6 and IL-10 are disease severity predictors. *Emerg Microbes Infect*. 2020;9(1):1123-1130. doi:10.1080/22221751.2020.1770129

Remy, K.E., Mazer, M., Striker, D.A. et al. Severe immunosuppression and not a cytokine storm characterizes COVID-19 infections. *JCI Insight*. 2020;5(17):e140329. doi:10.1172/jci.insight.140329

Burgos-Blasco, B., Güemes-Villahoz, N., Santiago, J.L., Fernandez-Vigo, J.I., Espino-Paisán, L., Sarriá, B., García-Feijoo, J., Martinez-de-la-Casa, J.M. (2020). Hypercytokinemia in COVID-19: Tear cytokine profile in hospitalized COVID-19 patients. Experimental Eye Research, 200. <https://doi.org/10.1016/j.exer.2020.108253>

Chen, G., Wu, D., Guo, W., Cao, Y., Huang, D., Wang, H., Wang, T., Zhang, X., Chen, H., Yu, H., Zhang, X., Zhang, M., Wu, S., Song, J., Chen, T., Han, M., Li, S., Luo, X., Zhao, J., & Ning, Q. (2020). Clinical and immunological features of severe and moderate coronavirus disease 2019. Journal of Clinical Investigation, 130(5), 2620–2629. <https://doi.org/10.1172/JCI137244>

Ghazavi, A., Ganji, A., Keshavarzian, N., Rabiemajd, S., & Mosayebi, G. (2020). Cytokine profile and disease severity in patients with COVID-19. Cytokine, 137. <https://doi.org/10.1016/j.cyto.2020.155323>

Guan, J., Wei, X., Qin, S., Liu, X., Jiang, Y., Chen, Y., Chen, Y., Lu, H., Qian, J., Wang, Z., Lin, X. (2020). Continuous tracking of COVID-19 patients’ immune status. International Immunopharmacology, 89, 1–11. <https://doi.org/10.1016/j.intimp.2020.107034>

Lin, L., Luo, S., Qin, R., Yang, M., Wang, X., Yang, Q., Zhang, Y., Wang, Q., Zhu, R., Fan, H., Wang, H., Hu, Y., Wang, L., Hu, D. (2020). Long-term infection of SARS-CoV-2 changed the body’s immune status. Clinical Immunology, 218. <https://doi.org/10.1016/j.clim.2020.108524>

Luo, W., Zhang, J. W., Zhang, W., Lin, Y. L., & Wang, Q. (2020). Circulating levels of IL-2, IL-4, TNF-α, IFN-γ, and C-reactive protein are not associated with severity of COVID-19 symptoms. Journal of Medical Virology, 1–3. <https://doi.org/10.1002/jmv.26156>

Sugiyama, M., Kinoshita, N., Ide, S., Nomoto, H., Nakamoto, T., Saito, S., et al (2020). Serum CCL17 level becomes a predictive marker to distinguish between mild/moderate and severe/critical disease in patients with COVID-19. Gene, 766. <https://doi.org/10.1016/j.gene.2020.145145>

Tomic´ S, Đokic´ J, Stevanovic´ D, Ilic´ N, Gruden-Movsesijan A, Dinic´ M, Radojevic´ D, Bekic´ M, Mitrovic´ N, Tomasˇ evic´ R, Mikic´ D, Stojanovic´ D and Cˇ olic´ M (2021) Reduced Expression of Autophagy Markers and Expansion of Myeloid-Derived Suppressor Cells Correlate With Poor T Cell Response in Severe COVID-19 Patients. Front. Immunol. 12:614599. doi: 10.3389/fimmu.2021.614599

Ghazavi, A., Ganji, A., Keshavarzian, N., Rabiemajd, S., & Mosayebi, G. (2021). Cytokine profile and disease severity in patients with COVID-19. *Cytokine*, *137*, 155323. <https://doi.org/10.1016/j.cyto.2020.155323>

### No comparison between mild and severe

Lei, X., Dong, X., Ma, R. *et al.* Activation and evasion of type I interferon responses by SARS-CoV-2. *Nat Commun* **11,**3810 (2020). <https://doi.org/10.1038/s41467-020-17665-9>

Yao, Z., Zheng, Z., Wu, K., Junhua, Z. Immune environment modulation in pneumonia patients caused by coronavirus: SARS-CoV, MERS-CoV and SARS-CoV-2. *Aging (Albany NY)*. 2020;12(9):7639-7651. doi:10.18632/aging.103101

Bastard, P., Rosen, L.B., Zhang, Q., Michailidis, E., Hoffmann, H. et al (2020). Auto-antibodies against type I IFNs in patients with life-threatening COVID-19. Science, 370(423). <https://doi.org/10.1126/science.abd4585>

Antonelli, G., Turriziani, O., Pierangeli, A. et al. Type I interferons can be detected in respiratory swabs from SARS-Cov-2 infected patients. *J Clin Virol*. 2020;128:104450. doi:10.1016/j.jcv.2020.104450

Nguyen et al. Influenza, but not SARS-CoV-2, infection induces a rapid interferon response that wanes with age and diminished tissue-resident memory CD8+ T cells. Clinical & Translational Immunology (2021); e1242. doi: 10.1002/cti2.1242

Bénard, A., Jacobsen, A., Brunner, M. *et al.* Interleukin-3 is a predictive marker for severity and outcome during SARS-CoV-2 infections. *Nat Commun* **12,**1112 (2021). https://doi.org/10.1038/s41467-021-21310-4

### Compare before and after

Schultheiß, C., Paschold, L., Simnica, D. et al. Next-Generation Sequencing of T and B Cell Receptor Repertoires from COVID-19 Patients Showed Signatures Associated with Severity of Disease. *Immunity*. 2020;53(2):442-455.e4. doi:10.1016/j.immuni.2020.06.024

### Compare healthy and COVID-19

Petrey, A.C., Qeadan, F., Middleton, E. A., Pinchuk, I. V., Campbell, R. A., Beswick, E. J. (2020). Cytokine release syndrome in COVID-19: Innate immune, vascular, and platelet pathogenic factors differ in severity of disease and sex. Journal of Leukocyte Biology. <https://doi.org/10.1002/JLB.3COVA0820-410RRR>

### Compare during the course of the disease

Yan, Q., Li, P., Ye, X., Huang, X., Mo, X. et al. (2020). Longitudinal peripheral blood transcriptional analysis of COVID-19 patients captures disease progression and reveals potential biomarkers. m*edRxiv*. <https://doi.org/10.1101/2020.05.05.20091355>

WEI, L., MING, S., ZOU, B., WU, Y., HONG. Z. et al. Viral Invasion and Type I Interferon Response Characterize the Immunophenotypes during COVID-19 Infection (2020). Available at SSRN: <https://ssrn.com/abstract=3555695> or [http://dx.doi.org/10.2139/ssrn.3555695](https://dx.doi.org/10.2139/ssrn.3555695)

### No measuring of IFN

Berenguer, J. et al., Characteristics and predictors of death among 4035 consecutively hospitalized patients with COVID-19 in Spain, *Clinical Microbiology and Infection*, <https://doi.org/10.1016/j.cmi.2020.07.024>

Du, Y., Tu, L., Zhu, P. et al. Clinical Features of 85 Fatal Cases of COVID-19 from Wuhan. A Retrospective Observational Study. *Am J Respir Crit Care Med*. 2020;201(11):1372-1379. doi:10.1164/rccm.202003-0543OC

Yang, Y., Shen, C., Li, J. et al. Plasma IP-10 and MCP-3 levels are highly associated with disease severity and predict the progression of COVID-19. *J Allergy Clin Immunol*. 2020;146(1):119-127.e4. doi:10.1016/j.jaci.2020.04.027

Bost, P., Giladi, A., Liu, Y., Bendjelal, Y., Xu, G., David, E., Blecher-Gonen, R., Cohen, M., Medaglia, C., Li, H., Deczkowska, A., Zhang, S., Schwikowski, B., Zhang, Z., & Amit, I. (2020). Host-Viral Infection Maps Reveal Signatures of Severe COVID-19 Patients. *Cell*, 181(7). <https://doi.org/10.1016/j.cell.2020.05.006>

### Comments

Thoutam, A., Breitzig, M., Lockey, R. *et al.* Coronavirus: a shift in focus away from IFN response and towards other inflammatory targets. *J. Cell Commun. Signal.* **14,**469–470 (2020). <https://doi.org/10.1007/s12079-020-00574-3>

Acharya, D., Liu, G. & Gack, M.U. Dysregulation of type I interferon responses in COVID-19. *Nat Rev Immunol* **20,**397–398 (2020). <https://doi.org/10.1038/s41577-020-0346-x>

Wang, Z., Pan, H. & Jiang, B. Type I IFN deficiency: an immunological characteristic of severe COVID-19 patients. *Sig Transduct Target Ther* **5,**198 (2020). <https://doi.org/10.1038/s41392-020-00306-4>

### Interferon type not specified

Wan, S., Yi, Q., Fan, S., Lv, J., Zhang, X., Guo, L., Lang, C., Xiao, Q., Xiao, K., Yi, Z., Qiang, M., Xiang, J., Zhang, B., & Chen, Y. (2020). Characteristics of lymphocyte subsets and cytokines in peripheral blood of 123 hospitalized patients with 2019 novel coronavirus pneumonia (NCP). *medRxiv*. <https://doi.org/10.1101/2020.02.10.20021832>

### Insufficient data

Trouillet-Assant, S., Viel, S., Gaymard, A., Pons, S., Richard, J.-C., Perret, M., Villard, M., Brengel-Pesce, K., Lina, B., Mezidi, M., Bitker, L., & Belot, A. (2020). Type I IFN immunoprofiling in COVID-19 patients. *J Allergy Clin Immunol*, 146(1), 206–208.

## Gene expression

Huang, L., Shi, Y., Gong, B., Jiang, L., Liu, X., & Yang, J. (2020). Blood single cell immune profiling reveals the interferon-MAPK pathway mediated adaptive immune response for COVID-19. *medRxiv*. doi: <https://doi.org/10.1101/2020.03.15.20033472>

Saichi, M., Ladjemi, M. Z., Korniotis, S., & Rousseau, C. (2020). Single cell RNA sequencing of blood antigen-presenting cells in severe Covid-19 reveals multi-process defects in antiviral immunity. MedRxiv. **doi:** https://doi.org/10.1101/2020.07.20.212837

Lee, J. S., Park, S., Jeong, H. W., Ahn, J. Y., Choi, S. J., Lee, H., Choi, B., Nam, S. K., Sa, M., Kwon, J. S., Jeong, S. J., Lee, H. K., Park, S. H., Park, S. H., Choi, J. Y., Kim, S. H., Jung, I., & Shin, E. C. (2020). Immunophenotyping of covid-19 and influenza highlights the role of type i interferons in development of severe covid-19. *Science Immunology*, 5(49), 1–16. <https://doi.org/10.1126/sciimmunol.abd1554>

Wu, P. Chen, D. Ding, W., Wu, P., & Hou, H. (2020). The Trans-omics Landscape of COVID-19. *medRxiv*. doi: <https://doi.org/10.1101/2020.07.17.20155150>

Yao, C., Bora, S. A., Parimon, T., Zaman, T., Friedman, O. A., Palatinus, J. A., Surapaneni, N. S., Matusov, Y. P., Chiang, G. C., Kassar, A. G., Patel, N., Green, C. E., Aziz, A. W., Suri, H., Suda, J., Lopez, A. A., Martins, G. A., Stripp, B. R., Gharib, S. A., … Chen, P. (2020). Cell type-specific immune dysregulation in severely ill COVID-19 patients. *medRxiv*. https://doi.org/10.1101/2020.07.23.20161182

Schulte-Schrepping, J., Reusch, N., Paclik, D., Baßler, K., Schlickeiser, S. et al. (2020). Severe COVID-19 Is Marked by a Dysregulated Myeloid Cell Compartment. *Cell*, 182(6), 1419–1440. <https://doi.org/10.1016/j.cell.2020.08.001>

Zhang, J., Wang, X., Xing, X. *et al.* Single-cell landscape of immunological responses in patients with COVID-19. *Nat Immunol* **21,**1107–1118 (2020). <https://doi.org/10.1038/s41590-020-0762-x>

Lin JW, Tang C, Wei HC, et al. Genomic monitoring of SARS-CoV-2 uncovers an Nsp1 deletion variant that modulates type I interferon response. *Cell Host Microbe*. 2021;29(3):489-502.e8. doi:10.1016/j.chom.2021.01.015

Sadanandam et al. A blood transcriptome-based analysis of disease progression, immune regulation, and symptoms in coronavirus-infected patients. Cell Death Discovery (2020) 6:141 <https://doi.org/10.1038/s41420-020-00376->
